# Falls prevention in community-dwelling older adults and implementation of world falls guidelines: a call for action across Europe by the European Geriatric Medicine Society Special Interest Group on Falls and Fractures

**DOI:** 10.1007/s41999-025-01206-y

**Published:** 2025-06-12

**Authors:** Nathalie van der Velde, Lotta J. Seppala, Alvaro Casas Herrero, Cedric Annweiler, Anna Björg Jónsdóttir, Hubert Blain, Yannis Dionyssiotis, Sofia Duque, James Frith, Bahaa N. Francis, Tomasz Grodzicki, Alison Hay, Luisa Alejandra Hernández-Sánchez, Roope Jaatinen, Christof J. Kadane, Marko Kolsek, Tomasz Kostka, Lisa McGarrigle, Stany Perkisas, Christoph Pertinatsch, Beatrice Pettersson, Jean-Yves Reginster, Carmelinda Ruggiero, Opinder Sahota, François-Xavier Sibille, Balamrit Singh Sokhal, Dawn A. Skelton, Joke Spildooren, Emma Stanmore, Hana Vankova, Ellen Vlaeyen, Yelda Ozturk, Ozlem Yilmaz, Chris Todd, Caterina Trevisan, Hanna Willems, Manuel Montero-Odasso, Finbarr C. Martin, Jesper Ryg, Tahir Masud

**Affiliations:** 1 Internal Medicine, Section of Geriatric Medicine, Amsterdam UMC Location University of Amsterdam, Amsterdam, The Netherlands; 2 Amsterdam Public Health Research Institute, Amsterdam, the Netherlands; 3 Geriatrics Department, Universitario de Navarra (HUN), C/Irunlarrea s/n 31008, Pamplona, Spain; 4 Navarra Institute for Health Research (IdiSNA), Pamplona, Spain; 5 CIBER of Frailty and Healthy Aging (CIBERFES), Instituto de Salud Carlos III, Madrid, Spain; 6 UNIV ANGERS, UPRES EA 4638, University of Angers, Angers, France; 7 Department of Geriatric Medicine and Rehabilitation, Research Center On Autonomy and Longevity, University Hospital, Angers, France; 8 Department of Medical Biophysics, Schulich School of Medicine and Dentistry, Robarts Research Institute, The University of Western Ontario, London, ON Canada; 9 Department of Geriatric Medicine, University Hospital of Iceland, Reykjavík, Iceland; 10 Faculty of Medicine, University of Iceland, Reykjavík, Iceland; 11 Geriatric Department, Montpellier University, Montpellier, France; 12 2nd Physical Medicine and Rehabilitation Department, National Rehabilitation Center EKA, Athens, Greece; 13 Preventive Medicine and Public Health Institute, Faculty of Medicine, University of Lisbon, Hospital Cuf Descobertas, Lisbon, Portugal; 14 Population Health Sciences Institute, Newcastle University, Newcastle Upon Tyne, UK; 15 Geriatric Division, Holy Family Hospital, Bar Ilan University, Nazareth, Israel; 16 Department of Internal Medicine and Gerontology, Jagiellonian University Medical College, Kraków, Poland; 17 Department of Emergency Medicine, Wexford General Hospital, Wexford, Ireland; 18 Servicio de Geriatría, Hospital Universitario Ramón y Cajal (IRYCIS), Madrid, Spain; 19 Oulunkylä Rehabilitation Centre, Helsinki, Finland; 20 University of Helsinki, Helsinki, Finland; 21 Department for Scientific Research in Rehabilitation, Pension Insurance Austria, Vienna, Austria; 22 Research Unit for Aging and Life Long Health, Medical University of Graz, Graz, Austria; 23 Department of Family Medicine, Faculty of Medicine, University of Ljubljana, Korytkova ulica 2, Ljubljana, Slovenia; 24 Department of Geriatrics, Healthy Ageing Research Centre (HARC), Medical University of Lodz, Pomorska 247/249, 92-209 Łódź, Poland; 25 National Institute for Health and Care Research (NIHR) Policy Research Unit in Healthy Ageing, School of Health Sciences, Faculty of Biology, Medicine and Health, The University of Manchester, Manchester, M13 9PL UK; 26 School of Health Sciences, Faculty of Biology, Medicine and Health, The University of Manchester, Manchester, M13 9PL UK; 27 Manchester Academic Health Science Centre, Manchester, M13 9NQ UK; 28 University Center for Geriatrics, University of Antwerp/ZAS, Antwerp, Belgium; 29 Department of Community Medicine and Rehabilitation, Umeå University, Umeå, Sweden; 30 Protein Research Chair, Biochemistry Dept, College of Science, King Saud University, Riyadh, Kingdom of Saudi Arabia; 31 Gerontology and Geriatric Section, Department of Medicine and Surgery, University of Perugia, Perugia, Italy; 32 Department of Healthcare of Older People, Nottingham University Hospitals NHS Trust, Nottingham, NG72UH UK; 33 Department of Trauma and Orthopaedics, Nottingham University Hospitals NHS Trust, Nottingham, UK NG72UH; 34 Geriatric Medicine Departement, CHU UCL Namur, Yvoir, Belgium; 35 Institute of Health and Society and Clinical Pharmacy and clinical Pharmacoepidemiology Research Group, Louvain Drug Research Institute Université Catholique de Louvain, Brussels, Belgium; 36 School of Medicine, Keele University, Newcastle-Under-Lyme, UK; 37 Royal Stoke University Hospital, University Hospitals North Midlands, Stoke-On-Trent, UK; 38 School of Health and Life Sciences, Glasgow Caledonian University, Glasgow, UK; 39 Faculty of Rehabilitation Sciences, REVAL-Rehabilitation Research Centre, Hasselt University, Diepenbeek, Belgium; 40 Manchester University NHS Foundation Trust, Manchester, M13 9WL UK; 41 Cooperatio 34, Department of Internal Medicine, Third Faculty of Medicine, Charles University, Prague, Czech Republic; 42 University Hospital Královské Vinohrady, Prague, Czech Republic; 43 Faculty of Medicine and Life Sciences, Hasselt University, Diepenbeek, Belgium; 44 Department of Public Health and Primary Care Academic Centre for Nursing and Midwifery, KU Leuven, Leuven, Belgium; 45 Department of Internal Medicine, Division of Geriatrics, TR Ministry of Health Eskisehir City Hospital, Eskisehir, Turkey; 46 Division of Geriatric Medicine, Department of Internal Medicine, Istanbul Training and Research Hospital, Samatya, Istanbul, Turkey; 47 National Institute for Health and Care Research, Applied Research Collaboration-Greater Manchester, School of Health Sciences, Faculty of Biology, Medicine and Health, Manchester, M13 9PL UK; 48 Department of Medical Science, University of Ferrara, Ferrara, Italy; 49 Gait and Brain Lab, Parkwood Institute and Lawson Health Research Institute, London, ON Canada; 50 Schulich School of Medicine & Dentistry, Department of Medicine and Division of Geriatric Medicine, University of Western Ontario, London, ON Canada; 51 Department of Epidemiology and Biostatistics, University of Western Ontario, London, Canada ON; 52 Population Health Sciences, Faculty of Life Sciences and Medicine, King’s College London, London, SE1 9RT UK; 53 Geriatric Research Unit, Department of Geriatric Medicine, Odense University Hospital, Odense, Denmark; 54 Department of Clinical Research, University of Southern Denmark, Odense, Denmark; 55 Nottingham University Hospitals NHS Trust, Nottingham, UK

**Keywords:** Accidental falls, Implementation, World falls guidelines, Older adults

## Abstract

Falls among older adults represent a significant public health challenge due to their consequences, including serious injuries, increased morbidity and mortality, decreased quality of life, and heightened healthcare costs. The World Falls Guidelines (WFG), published in 2022, offer a robust framework for evidence-based interventions; however, the uptake of these guidelines into clinical practice across Europe remains inconsistent. Key barriers to implementation include insufficient resources, a lack of trained healthcare professionals, and limited integration into existing healthcare systems. This position paper by the EuGMS Special Interest Group (SIG) on Falls and Fractures addresses the implementation of the WFG among community-dwelling older adults and falls prevention across Europe by providing an overview of the current status of WFG adoption in Europe and discusses the challenges and opportunities for implementation. We provide an overview of the current resources to support the clinical practice of falls prevention, implementation guides, and educational programs. Additionally, we discuss what is necessary for the future development of these resources and for advancing research. The EuGMS SIG on Falls and Fractures advocates for a commitment of healthcare providers as well as insurers, policymakers, and other stakeholders to collaborative European initiatives—such as developing a standardised falls prevention strategy, promoting evidence-based implementation plans, establishing a European-wide research agenda, and creating under- and postgraduate curricula—which are essential for advancing falls prevention efforts across Europe.

**Supplementary Information:**

The online version contains supplementary material available at 10.1007/s41999-025-01206-y.

## Key summary points

**Aim:**

To formulate strategic recommendations for enhancing the implementation of the World Falls Guidelines among community-dwelling older adults and improve falls prevention efforts across Europe.

**Findings:**

Effective fall prevention policies require a comprehensive multifaceted approach, which includes the integration of falls prevention strategies into broader healthcare policies, public health messaging and the development of a comprehensive curriculum in healthcare education. Design and delivery of services should include community participation involving older adults with lived falls experience and the application of implementation science. Establishing a standardized, locally adaptable European toolkit for falls prevention plans along with tailored implementation tactics, could effectively address barriers to the successful implementation.

**Message:**

A collaborative commitment of relevant stakeholders to European initiatives—such as developing a standardized falls prevention strategy, promoting evidence-based implementation plans, establishing a European-wide research agenda, and creating an under- and postgraduate curriculum—is essential for advancing falls prevention efforts across Europe.

**Supplementary Information:**

The online version contains supplementary material available at 10.1007/s41999-025-01206-y.

## Introduction

Falls represent a significant public health concern both globally and in Europe, being the second leading cause of accidental or unintentional injury deaths worldwide, with approximately 684,000 fatalities annually, according to the World Health Organization (WHO) [1]. Moreover, 172 million people suffer short- or long-term disability as a result of falls [1]. The impact of falls is particularly severe for older adults, as falls are the primary cause of fatal injuries in older adults and negatively affect functional independence and quality of life [2–4]. Furthermore, falls are among the most preventable causes of visits to emergency departments, hospital admissions, and transitions to nursing homes [5]. Besides their human cost, falls place a significant financial burden on healthcare systems, with up to 1.5% of total medical expenditures linked to fall-related care [6].

The first World Guidelines for Falls Prevention and Management for Older Adults (WFG) (2022) were developed in response to the critical global and European challenge posed by the increasing incidence of falls and associated injuries [7]. Created by the World Falls Guidelines Task Force, these guidelines originally involved 96 multidisciplinary experts from 39 countries and 36 scientific and academic societies. They provide evidence-based recommendations that aim to reduce the risk of falls in older people, recognising that while falls cannot be completely prevented, these strategies significantly lower the likelihood of falls and improve outcomes [7]. Key messages of the guidelines included: (i) conduct risk assessment and stratification; (ii) provide general recommendations to optimise physical function and mobility for all older adults; and (iii) offer a holistic, multidomain intervention for older adults at high risk of falls, taking into account their priorities, beliefs, and available resources [7].

There is notable variation in the implementation of the WFG across European countries, ranging from minimal actions to full integration into national recommendations and guidelines, as illustrated in Table 1. The data presented in the table was provided by the co-authors from various countries and reflects their best knowledge of the current state of WFG adoption and implementation in their respective healthcare systems.

**Table 1:** Implementation status of WFG in selected European countries

Country	Current status
Austria	The summary of the guidelines has been translated to German [8] by German authors. There is no national guideline for fall prevention in Austria based on the World Falls Guidelines. There are small regional initiatives that promote fall prevention
Belgium	The WFG has been integrated in the local Flemish (Belgian) guideline for nursing homes. This guideline is validated by CEBAM (Belgian Centre for Evidence-Based Medicine/Cochrane Belgium). Based on this guideline, new materials have been and will be further developed. This information is freely available on the website of the Centre of Expertise for Fall and Fracture Prevention Flanders [9] and more in detail [10, 11]
Czech Republic	A brief summary and algorithm was translated into Czech. The translation is used for education of medical students at Charles University and referred in a monography (Vankova H: Geriatrie in Rychlik J., Widimsky P. Vnitřní lékařství). The brief narrative summary was published in Czech [12] and referred in a review on the topic [13]. The WFG or its components have not yet been integrated into national/regional guidelines or health policies on falls prevention. Czech Society of Gerontology and Geriatric is collaborating with the Czech Ministry of Health on competition of National Plan to promote geriatrics, the global governmental vision of needs of geriatric citizens and support of geriatric services. This National plan includes the initiative regarding falls prevention. This initiative is to be followed by detailed implementation plans including WFG implementation
Denmark	The algorithm has been partially translated, but this effort has been done locally. There are no national guidelines for falls prevention in place. A special interest group under the Danish Geriatric Society is working on translating and implementing the WFG within geriatrician-led falls clinics. These efforts are driven with no governmental involvement or financial support
Finland	A national institute for health promotion research (UKK Instituutti) has translated the algorithm and recommendations into both primary languages (Finnish, Swedish) [14]. The WFG have been integrated into earlier national recommendations. Distribution of the information has been adopted by the National Institute of Health and Welfare [15]. The WFG have been integrated into an electronic platform available for all [16] as well as the web-based archive accessed only by healthcare professionals (Terveysportti)
France	A French version of the main points, practical messages, and of the algorithm of the WFG has been published in French [17]. The French guidelines (Haute Autorité de Santé) on falls prevention are from 2005. An SFGG special interest group on falls and fracture prevention was been set up in November 2024. One of the missions of this SIG will be to propose to the Haute Aurorité de santé to update the French guidelines, based on WFG. A French Falls prevention plan was launched in 2022 [18], i.e. before the publication of the WFG. Different French areas are pilot areas to implement the plan. Each region (each region health agency) has a specific project and the best initiatives will serve as models for a national dissemination. In the south and west part of Montpellier, the Occitanie Health agency supports a project aiming to implement the WFG by a community of health professionals (2025–2027 project)
Greece	The WFG has not been translated into Greek. It seems that neither the WFG nor any of its components have been incorporated into national/regional public health guidelines or policies on falls prevention
Iceland	The WFG has not been translated into Icelandic. It is not customary to translate clinical guidelines in Iceland. There are no national or regional guidelines for falls prevention in Iceland. However, individual healthcare institutions, such as Landspitali University Hospital and Heilsugæsla höfuðborgarsvæðisins (primary care centres in the capital region), have partially adopted the components of the WFG
Ireland	The WFG has not been integrated into national or regional guidelines. There is a policy on falls prevention from the Health Service Executive (HSE), but it has not been updated to include the WFG
Israel	The WFG has not been translated into Hebrew or Arabic. It seems that neither the WFG nor any of its components have been incorporated into national/regional public health guidelines or policies on falls prevention
Italy	The WFG has not been translated into Italian. The WFG has not been formally adopted by Italian health authorities. Existing national guidelines share similar objectives, such as assessing fall risk and implementing preventive interventions
Netherlands	The WFG algorithm has been translated by VeiligheidNL [19]. The Dutch guidelines follow a modular update structure [20]. Each module under revision considers the findings and recommendations of the WFG, evaluating them against the existing literature and the local context. Where applicable, recommendations are either adapted or modified to suit the Dutch healthcare environment. A module of assessment of fall-risk-increasing drugs has been adapted and authorised. Another module (falls as an atypical disease presentation or underlying diseases) is in the authorisation phase. Four additional modules (fall risk stratification and assessment, falls preventive intervention community, falls preventive interventions hospital, falls preventive interventions nursing homes) are in the first phase of update. National Government has a falls prevention programme [21] that also supports WFG implementation including working implementation of the WFG algorithm, implementation of falls prevention strategies for low- and intermediate-risk groups, aligned with WFG recommendations, establishing financial structures within the community care to sustain these efforts, and coordinating discussions with stakeholders on implementing primary care-led and transmural approaches for assessing and treating high-risk patients
Poland	The WFG has not been translated into Polish. It seems that neither the WFG nor any of its components have been incorporated into national/regional public health guidelines or policies on falls prevention
Portugal	The WFG has not been translated into Portuguese. WFG has not been integrated into national/regional guidelines or health policies on falls prevention
Slovenia	It seems that neither the WFG nor any of its components have been incorporated into national/regional public health guidelines or policies on falls prevention. A key messages of the guidelines has been translated as a part of national project 'Integration of geriatric management of the older persons' which includes training of health and social workers and other professionals working with older persons [22]
Spain	WFG has not yet been translated, but there is an ongoing effort to adapt them, alongside the Fragility approach (2022) [23]. Most regions are working toward incorporating the WFG into their policies, with documents expected to be available in 2025
Sweden	No national initiatives are currently underway to translate the WFG, or no formal integrations of the WFG into national or regional guidelines or health policies regarding fall prevention have been implemented. However, as a regional initiative, an educational platform has been developed both for healthcare professionals and individuals aged 65 and older. This initiative is a development and research project built on co-creation with social services, healthcare, and senior citizen organisations in the Region of Sörmland. The content on the platform is based on the WFG (algorithm, assessments, and interventions), with adjustments to align with nationally and regionally available resources and includes also fall prevention management and recommendations for individuals living in residential care facilities [24]
Turkey	The WFG has not been translated into Turkish. WFG has not been integrated into national/regional guidelines or health policies on falls prevention
UK	The National Institute for Health and Care Excellence (NICE) published national guidance for healthcare professionals and commissioners that covers assessment of fall risk and interventions to prevent falls in people aged 65 and over [25]. The NICE guidance is currently being updated and is due for publication in March 2025. The draft [26] does not mention the WFG. Although the draft guideline does not recommend a “falls risk assessment”, the sequence of questions and assessment of gait and balance are the same as the algorithm in the WFG, albeit no threshold of gait and balance assessment is suggested. The range of assessments and interventions is also same or very similar. The national policy and commissioning bodies do not specifically recommend following the WFG, but do recommend falls prevention/falls risk reduction activities. There is no one organisation having statutory responsibility for commissioning fall prevention programmes in the UK British Geriatrics Society Falls and Bone Health Special Interest Group, with annual conferences, have been supporting implementation of the WFG [27]

However, the implementation of the WFG in clinical practice worldwide and across Europe is hindered by several challenges. A recent systematic review reported that the availability of necessary resources is the most frequently stated determinant influencing the implementation of fall prevention programmes in a community [28]. This challenge is evident across all phases of fall prevention, from risk stratification to the effective delivery and ongoing evaluation of these interventions. Other commonly reported factors include knowledge, intentions/beliefs, and motivation at both the level of older adults and healthcare professionals, the integration of interventions into existing practices, communication, team and referral processes, and financial (dis)incentives [28]. These challenges were further highlighted by two recent large, pragmatic trials on multidomain fall prevention interventions in a community, which lacked the expected preventive effect in terms of falls outcomes [29, 30]. Issues related to uptake, fidelity, and adherence to the interventions likely contributed, at least in part, to the lack of the effect observed [31].

This position paper by the EuGMS Special Interest Group (SIG) on Falls and Fractures discusses recommendations for the enhanced implementation of the WFG among community-dwelling older adults and effective fall prevention strategies in Europe. We focus on community-dwelling older adults and we do not address fall prevention in hospital or nursing home settings. Our focus is on improving current clinical practice, addressing challenges in implementation, underlining the role of education, and outlining research priorities. The recommendations are derived from a non-systematic review of existing literature combined with expert knowledge. The multidisciplinary expert group consisted of 39 members of the SIG Falls and Fractures from 19 countries. NvdV and LS authored the initial draft of the manuscript. All authors contributed to the refinement of the recommendations by providing critical feedback, which was discussed during an online meeting. Additionally, members shared both international and national resources relevant to clinical practice, implementation, and education.

## Clinical practice

**Fig. 1:**
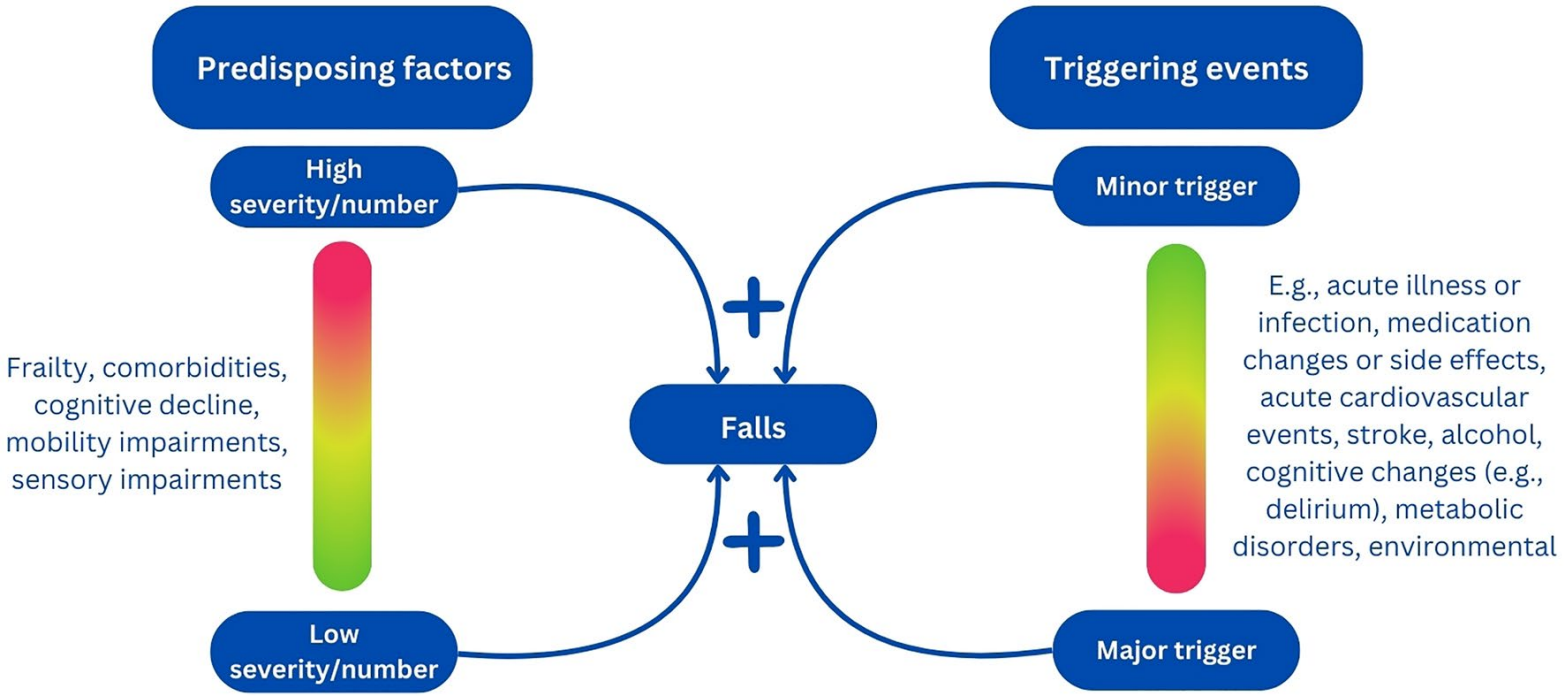
Multifactorial model for falls

Falls, as any of the geriatric giants often serve as warning signs of underlying, undetected health issues such as acute and chronic diseases, deconditioning or adverse drug effects, and arise habitually from multifactorial aetiology. Similar to the multifactorial model for other geriatric giant delirium, developed by Inouye [32], when addressing fall risk, the full spectrum of vulnerability and precipitating factors needs to be considered [33]. Falls often result from the interaction of patient predisposing factors and triggering events (Fig. 1). Most falls occur when an individual is moving and happens when there is a change within the person, their behaviour (e.g. acute illness causing delirium), or their environment. The lower their pre-existing reserve, the more likely is that any (minor) trigger can result in a failure to maintain balance.

**Fig. 2:**
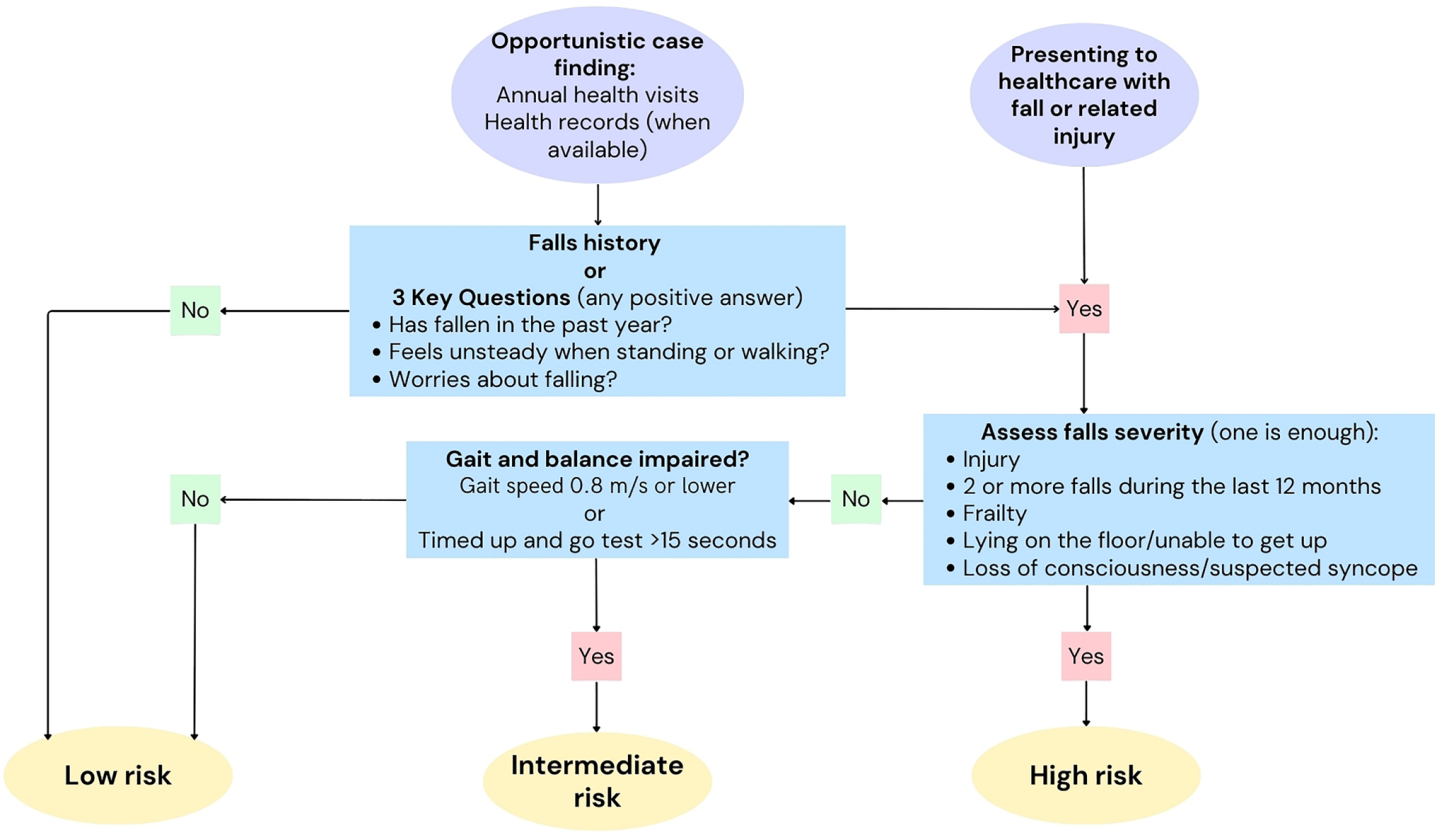
Algorithm for risk stratification for community-dwelling older adults adapted from the WFG [7]

**Fig. 3:**
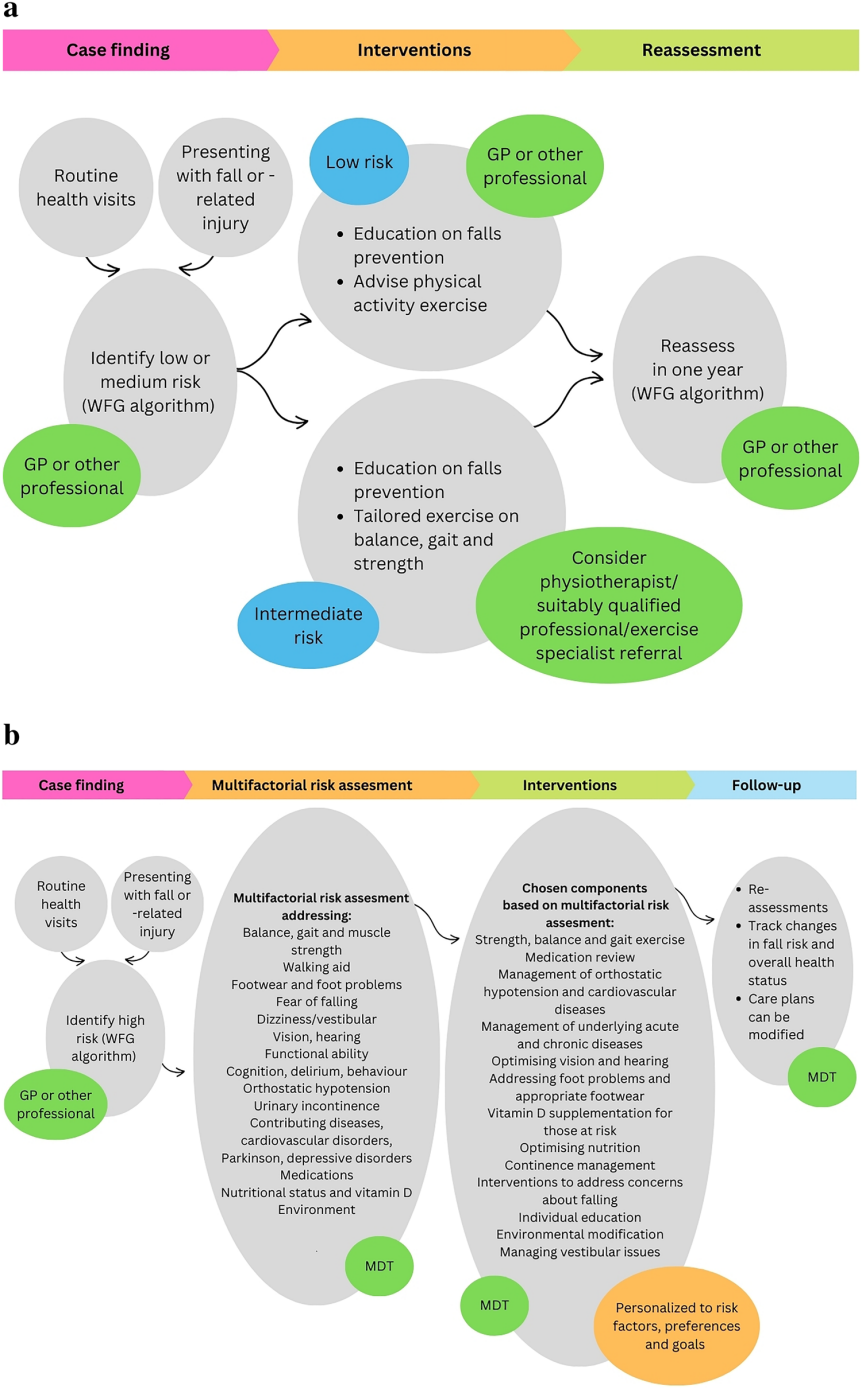
a Case finding, interventions, and reassessment for low- and intermediate-risk older adults adapted from the World Falls Guidelines. b Case finding, risk assessment, interventions, and follow-up for high-risk older adults adapted from the World Falls Guidelines. MDT = multidisciplinary team, GP = general practitioner

This understanding warrants a multifactorial approach to fall prevention for patients at high risk, emphasising the need to assess and address a comprehensive range of factors influencing each individual's risk. The factors contributing to recurrent falls can vary for each individual over time, making it essential to address all potential contributors as part of a comprehensive preventative approach and to readdress regularly. The WFG introduced a novel fall risk stratification algorithm for community-dwelling older adults to differentiate these high-risk individuals from low- and intermediate-risk older persons (Fig. 2) [7]. Older adults at low risk for falls should be provided with education on fall prevention and exercise for general health [34], if interested (Fig. 3a) [7]. Information should be communicated in a manner that is likely to influence behaviour effectively. Incorporating behaviour change techniques into physical activity (PA) interventions can help reinforce shifts in behaviour and attitudes. A recent review highlighted seven intervention components that significantly impact PA levels, including goal setting, personalised feedback, and both onsite and post-intervention support [35].Those at intermediate risk for falls should receive the same educational resources, along with targeted exercise recommendations or a referral to a physiotherapist or trained exercise instructor/clinical exercise physiologist to enhance balance, increase muscle strength, and as a result reduce their risk of falling (Fig. 3a) [7].

Frailty for risk stratification can be determined through previously identified frailty or a positive result on a validated detection tool including the frailty phenotype (FP) and the clinical frailty scale (CFS). The FP assesses five criteria—slow gait speed, low physical activity, unintentional weight loss, exhaustion, and muscle weakness—categorising individuals as frail (≥ 3 criteria), prefrail (1–2 criteria), or not frail (0 criteria). The CFS is a semi-quantitative scale ranging from 1 (very fit) to 9 (terminally ill), with a score of ≥ 4 indicating frailty.

For community-dwelling older people at high risk, the WFG recommends a multifactorial falls risk assessment to institute individualised and tailored interventions. However, multifactorial and personalised falls prevention programmes tailored to specific context are scarcely available in different European regions [36]. This important gap presents challenges that require the development of local, transmural, multidisciplinary falls prevention pathways tailored to resources available to support the recommendations of the WFG. The pathways should integrate case finding of high-risk individuals, fall risk assessment, evidence-based interventions, and effective follow-up (Fig. 3b). A multidisciplinary approach is crucial for effective fall prevention, as it ensures the integration of diverse expertise from healthcare professionals such as physicians, physiotherapists, occupational therapists, and nurses. The fall prevention pathways should create a strong connection between primary care and specialised fall prevention services such as falls clinics. This is crucial as fall prevention is complex, and specific expertise is generally lacking in primary care settings, which can hinder effective implementation and management. The distribution of tasks within the care pathways depends on the national context. The referral processes should be kept as simple as possible to ensure seamless transitions between the different stages—case finding, risk assessment, intervention, and follow-up. Simplifying these processes may help minimise the risk of drop-off between stages, ensuring that patients receive continuous and coordinated care throughout the entire fall prevention pathway.

Furthermore, a key consideration is that fall prevention should not occur in isolation in the community or specialised care; it should be integrated with other healthcare services crossed by older adults' care pathways including, e.g. emergency departments (ED), rehabilitation centres, or those focused on fragility fracture prevention. The earlier SIG Falls and Fractures position paper highlighted the importance of integrating the optimisation of bone health with the implementation of falls prevention measures in fracture prevention [37]. Furthermore, the ED is an opportune setting to intervene for patients who have fallen. While interventions in the ED may be challenging, they are crucial, as patients are already present, and there is a risk that they may not be referred for further intervention. For example, medication review has been proven feasible in the ED setting [38].

Furthermore, cross-sector cooperation is vital. Collaborative efforts between healthcare providers, social services, and community organisations not only support more holistic fall prevention strategies, but can among others also improve case-finding accuracy, ensure continuity of care, and lower barriers for older adults to participate in prevention programmes. Such collaboration allows for a continuous framework, making fall prevention accessible and responsive across diverse care levels and community services. This inclusive, integrated approach enhances the reach to vulnerable populations, especially those in underserved areas, by addressing fall prevention within a broad, supportive network.

A key goal for the future would be the creation of a European toolkit for a fall prevention pathway, designed to be adaptable to national and local contexts. This resource would provide a standardised, evidence-based approach while allowing flexibility for healthcare systems to tailor the pathway according to their specific needs and circumstances. Ideally, this toolkit would be supported by digital tools aimed at older adults who have experienced falls, caregivers, and healthcare professionals. These digital tools could be particularly beneficial for countries lacking consistent primary care structures, geriatric medicine expertise, or dedicated falls clinics. To support the current practice, we list important resources that can support the clinical practice of falls prevention (Table 2). National resources are listed in the Appendix (Supplementary Table 1). While not all resources may fully align with the WFG, they are included here due to their potential relevance. Furthermore, the Step Safely: Strategies to Prevent and Manage Falls Across the Life-Course, a technical package produced by the WHO, offers evidence-based strategies, practical and policy approaches, and implementation guidance for preventing falls and managing fall-related injuries across the life course, aimed at practitioners, policymakers, and advocates [1], and the ICOPE Handbook helps community health workers implement WHO’s Integrated Care for Older People (ICOPE) guidelines, offering pathways to address decline in mobility, malnutrition, visual impairment, hearing loss, cognitive decline, and depressive symptoms [39]. It promotes person-centred care to optimise intrinsic capacity and supports integrating preventive services into primary care for healthy ageing.

**Table 2:** Resources to support clinical practice of falls prevention in English

Resource	Description
Risk stratification for community-dwelling older adults	
WFG risk stratification algorithm for community-dwelling older adults	Categorises individuals into three groups: low, intermediate, and high fall risk [7]: Fig. 2
Risk assessment	
WFG	WFG includes a list of potential measurement instruments and approaches for multifactorial falls risk assessment (Table 4 in WFG) [7]
Comprehensive geriatric assessment (CGA) in primary care	The British Geriatrics Society (BGS) has developed an introduction for CGA in primary care [40]
*Interventions*	
Strength and balance exercise	i. National Prudent Healthcare Falls Prevention Task Force (Wales) and Ageing Well in Wales Guidance for Recommending Exercise for Older People to Reduce Falls Risks [41] ii. Later Life Training—Guidance and support for commissioners of falls prevention exercise programmes [42] iii. Vivifrail exercise program in 12 languages [43] iv. Royal Osteoporosis Society Consensus Statement on Exercise for Osteoporosis—Strong, Steady and Straight [44] v. Royal Osteoporosis Society Exercise Resources for Strong, Steady and Straight [45] vi. Centre for Ageing Better—Raising the Bar on strength and balance: The importance of community-based provision [46] vii. International Exercise Recommendations in Older Adults (ICFSR): Expert Consensus Guidelines [34]
Medication optimisation	i. STOPPFall: consensus list of fall-risk-increasing drugs and deprescribing tool [47] ii. Series of clinical reviews on deprescribing dilemmas of patients at high risk of falls: a themed journal issue on (de)prescribing dilemmas in older, multimorbid adults with increased fall risk providing the practicing clinician with practical resources, tools, tips, and tricks for safe FRIDs (de)prescribing [48] iii. STEADI material about medications linked to fall risk and how to conduct a medication review [49] iv. National Falls Prevention Coordination Group document on medicines and falls providing information and guidance on medication review for people at risk of falls [50]
Management of orthostatic hypotension and cardiovascular disorders	i. 2018 ESC Guidelines for the diagnosis and management of syncope [51] ii. Resources from the BGS Cardiovascular SIG [52]
Management of underlying and acute diseases	i. BGS introduction for CGA in primary care [40] ii. BGS good practice guide on frailty (in acute settings and in community) [53] iii. BGS. Silver Book II. Quality care for older people with urgent care needs [54] iv. NICE guidelines on multimorbidity [55]
Fracture risk management	i. NICE Guidelines on osteoporosis [56] ii. The UK National Osteoporosis Guideline Group (NOGG) clinical guideline for the prevention and treatment of osteoporosis [57] iii. Osteoporosis Canada 2023 Guideline Update Group. Clinical practice guideline for management of osteoporosis and fracture prevention in Canada: 2023 update [58] iv. Core principles for fracture prevention: North American Consensus from the National Osteoporosis Foundation, Osteoporosis Canada, and Academia Nacional de Medicina de Mexico [59]
Vision and hearing optimisation	i. The college of optometrists: material on vision and falls [60] ii. National Falls Prevention Coordination Group—resources for optometrists [61] iii. WHO guidance on identifying declines in hearing and vision in integrated care for older people (ICOPE): guidance for person-centred assessment and pathways in primary care [39]
Foot problems and footwear management	Information about safe shoes and footcare by The Stay On Your Feet^®^ programme from Australia [62]
Vitamin D supplementation for those at risk of vitamin D deficiency	i. Role of vitamin D supplementation in the management of musculoskeletal diseases: update from the European Society of Clinical and Economical Aspects of Osteoporosis, Osteoarthritis and Musculoskeletal Diseases (ESCEO) working group [63] ii. IOF position statement: vitamin D recommendations for older adults [64] iii. Evaluation, treatment, and prevention of vitamin D deficiency: an endocrine society clinical practice guideline [65] iv. Clinician’s guide to prevention and treatment of osteoporosis [66]
Sarcopaenia	International Clinical Practice Guidelines for Sarcopenia (ICFSR): Screening, Diagnosis and Management [67]
Nutrition management	i. ESPEN practical guideline: Clinical nutrition and hydration in geriatrics [68] ii. BGS resources on nutrition [69]
Continence management	BGS resource on urinary incontinence management: CGA in Primary Care Settings: Patients presenting with urinary incontinence [70]
Interventions to address concerns about falling	Commentary: Why should clinical practitioners ask about their patients’ concerns about falling? [71]
Individual education	i. CDC developed the STEADI (Stopping Elderly Accidents, Deaths & Injuries) initiative which includes educational materials for older adults and caregiver’s [72] ii. Fall prevention screening tools, checklists, and information designed for the consumer developed by the NSW Fall Prevention and Healthy Ageing Network [73] iii. A guide produced by AgeUK for older adults on how they can reduce their risk of falling [74] iv. PROFOUND patient exercise and information leaflets in over 15 European Languages [75] v. NHS Inform: Preventing Falls (including videos and information on how to get up from the floor after a fall) [76] vi. FallsAssistant—online action planning for older people to help prevent falls [77]
Environmental modification	i. The Royal College of Occupational Therapists practice guideline: Occupational Therapy in the prevention and management of falls in adults [78] ii. HomeFAST Occupational Therapy Assessment [79] iii. A scoping review of fall hazards in the homes of older adults and development of a framework for assessment and intervention [80]
Technology	i. Digital technologies to prevent falls in people living with dementia or mild cognitive impairment: a rapid systematic overview of systematic reviews [81] ii. A rapid review of digital approaches to support the engagement of older adults in strength and balance exercise [82]
Vestibular interventions	i. Clinical Practice Guideline: Benign Paroxysmal Positional Vertigo [83] ii. Fall Risk Management in Audiology and ENT Practice: The Role of Cognitive, Vestibular, and Auditory Function [84]
Pain interventions	i. Evidence-based clinical practice guidelines on the management of pain in older people [85] ii. Review Pharmacological Pain Treatment in Older Persons [86]

To enhance fall prevention strategies, it is essential to explore and understand the various technologies that can support and automate tasks within this framework. Several areas of fall prevention can utilise technology, such as predictive and prescriptive analytics with big data, video monitoring and alarm systems, wearable sensors, exergaming and virtual reality, robotics for home environment assessments, and personal coaching [87]. In the past few years, there have been some promising developments in fall prevention technology. Case finding is resource-intensive, and opportunistic case finding is underdeveloped in primary care settings. Therefore, utilising routinely collected electronic health records to automate the identification of fall risk presents considerable potential for supporting the more efficient implementation of falls prevention interventions. In the UK, for example, the eFalls prediction model was developed to address this need [88]. It is important to evaluate how the integration of this model into existing systems would impact patient care and outcomes, ultimately streamlining processes and improving the effectiveness of fall prevention strategies. Currently, the eFalls tool is being tested as a case stratification approach in Greater Manchester. Another notable example is StandingTall, which offers personalised guided progressive exercises through remote delivery. The clinical effectiveness of StandingTall was demonstrated through a randomised controlled trial comparing it to a health education programme for community-dwelling older adults in Australia, and in an implementation study in Australia and UK, it showed promising results [89, 90]. Furthermore, in a recent smart ± step trial RCT, participants who did exergame training had a significantly lower rate of falls compared to the control group, while this was not the case in the group that did cognitive training [91]. Keep-On–Keep-Up is a personalised, NHS-approved App with strength and balance exercises and health literacy games to prevent physical decline and frailty and has been shown to be an acceptable and easy to use falls prevention intervention [92]. Another example for technology-based exercise interventions is the Safe Step application, in which older adults independently create their own exercise programme using a repository of evidence-based exercises [93]. Finally, the SNOWDROP trial introduces an AI-based clinical decision support system for general practitioners (GPs) offering personalised deprescribing advice, along with a patient portal for patients with a high fall risk showed promising results by enhancing shared decision-making and improving satisfaction with communication during a consultation [94].

## Implementation

The difficulties in implementing evidence-based recommendations are well documented across healthcare, and this includes multifactorial fall prevention and exercise programmes [31, 95]. In the context of the WFG, implementation involves translating the evidence-based recommendations for fall prevention into real-world healthcare settings. A recent systematic review categorised the determinants influencing the implementation of multifactorial falls risk assessment and multidomain interventions in community-dwelling older people into seven domains: (i) guideline factors, (ii) individual healthcare professional factors, (iii) patient factors, (iv) professional interactions, (v) incentives and resources, (vi) organisational change, and (vii) social, political, and legal factors, as outlined by the Comprehensive Integrated Checklist of Determinants of practice framework [28]. The availability of necessary resources was the most often reported determinant in the literature [28]. Other commonly reported determinants were knowledge, intention/beliefs, and motivation of older people and healthcare professionals, adopting intervention into current practice, communication, team and referral processes, and financial (dis)incentives [28]. In line with the results of the systematic review, a survey conducted by the EuGMS SIG Falls and Fractures among European healthcare professionals identified the top-five barriers to falls prevention: staffing issues, lack of time, older adults' non-adherence to recommended strategies, workload related to falls prevention, and prioritising other tasks [96]. Notably, these barriers exhibited significant variation at both regional and, even more so, country-specific levels.

Another recent systematic review described the implementation strategies used to implement multifactorial fall prevention interventions in the community [97]. The review found that studies primarily focused on implementation strategies at the level of older adults and HCPs, highlighting the importance of tailoring, raising awareness, and encouraging participation in the implementation process [97]. Studies addressing implementation strategies at the organisational, community, and policy/society levels emphasised the significance of providing technical assistance, actively engaging stakeholders and forming coalitions as key strategies [97]. A recent study conducted in Norway showed that a co-created implementation strategy was possible, doable, and easy to implement if it fostered consensus, involved multi-level and interdisciplinary collaboration, minimised perceived time usage through utilisation of existing areas for implementation activities, and had good facilitation and structure in the implementation strategy [98].

**Fig. 4:**
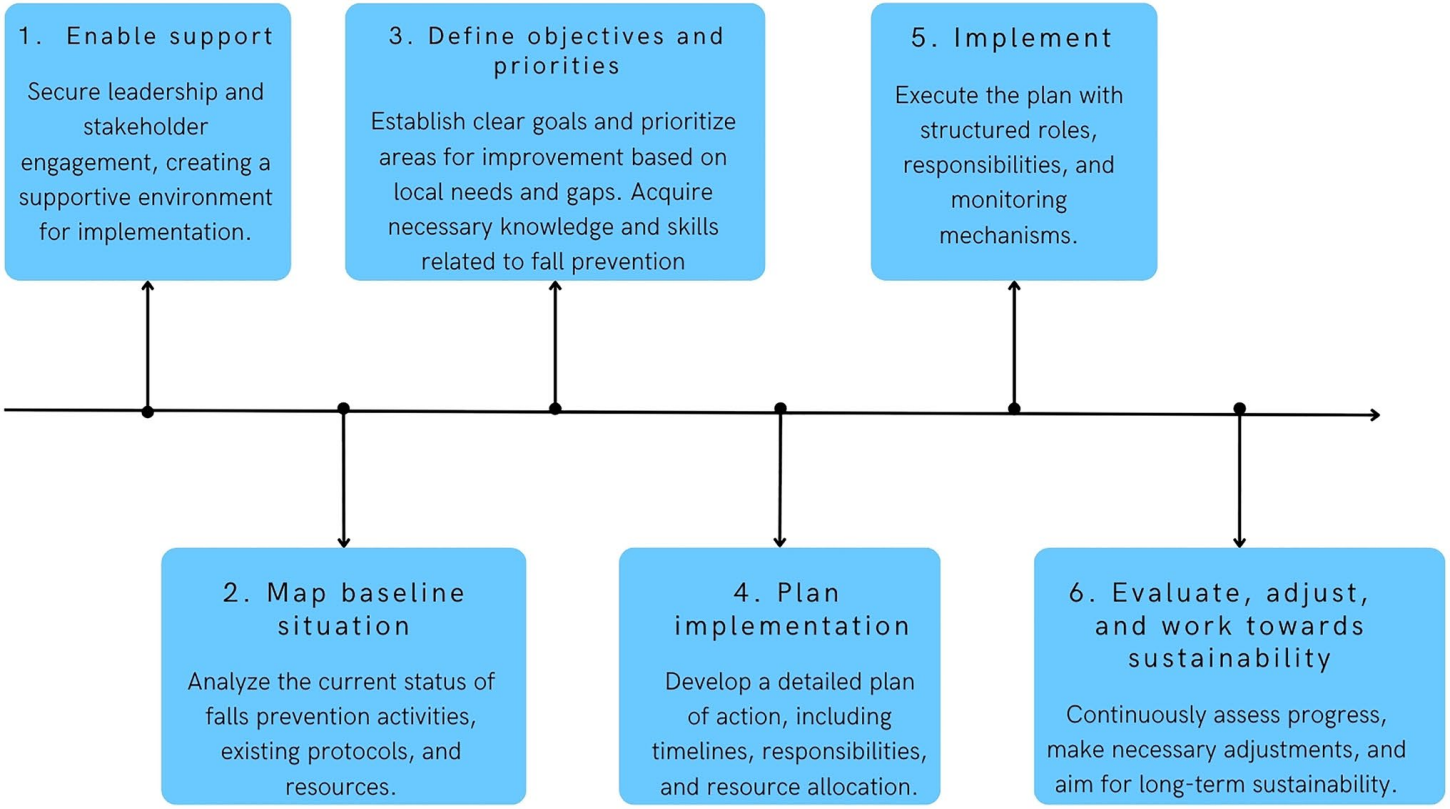
Steps for a local falls implementation plan [103]

To bridge the current gap between the evidence-based recommendations of the WFG and clinical practice, a local evidence-based implementation plan, tailored and (co)developed in collaboration with key stakeholders to address the specific context, is essential for effective falls prevention and management among older adults. We present the key principles for implementation in this position paper, but for optimal implementation, a more in-depth step-by-step European-wide guide adaptable to local context should be developed. Local adaptation is particularly important, as determinants affecting fall prevention such as limited financial resources, cultural hesitancy, or disparities in healthcare access are known to vary significantly between countries [31]. It is important to identify local clinic-, provider-, and patient-level barriers and facilitators in collaboration with stakeholders, including older adults, caregivers, local policymakers, and healthcare professionals [99]. Tailored implementation strategies should then be developed and systematically matched to these identified barriers [99]. Finally, pilot testing of these strategies will help determine their feasibility, acceptability, and appropriateness. Finally, pilot testing these strategies will help assess whether these strategies are feasible, acceptable, and appropriate [99]. A summary example of the steps that could be included in a local fall implementation plan is provided in Fig. 4.

National implementation guidance, where available, is listed in the Appendix (Supplementary Table 2). Also, the American Centers for Disease control and prevention (CDC) has developed A Guide to Implementing Effective Community-Based Fall Prevention Programs [100] and a Compendium of Effective Interventions [101]*.* The implementation guide provides organisations with the essential building blocks for creating successful fall prevention programmes, offering examples, resources, and practical tips. It outlines the necessary resources to implement and sustain fall prevention efforts, ensuring long-term effectiveness. For more specific components of fall prevention, implementation toolkits have been developed to assist in adopting evidence-based programmes into practice. One such example is the FaME (Falls Management Exercise) implementation toolkit, which provides all the necessary information to set up and run a FaME programme [102]. This comprehensive toolkit includes guidance on all aspects from creating the initial business case to effectively promoting the programme to participants.

## Education

A recent survey conducted by the SIG Falls and Fractures revealed widespread dissatisfaction with undergraduate education in falls prevention among all healthcare professionals, with only about a quarter of respondents agreeing or strongly agreeing that it adequately prepared them for clinical practice [96]. In 2014, the European undergraduate curriculum in geriatric medicine was published, outlining the minimum requirements that medical students should meet before graduating [104]. The curriculum states they should be able to describe the pathophysiology, diagnosis, assessment, management, and preventive strategies for common geriatric syndromes in older people, including falls and movement disorders [104]. However, there is a clear need to map what is currently being taught in medical schools across Europe regarding falls prevention. Additionally, an international undergraduate curriculum specifically focused on falls prevention should be developed to ensure more comprehensive training in this crucial area. In 2019, the European postgraduate curriculum in geriatric medicine was published, detailing the minimum recommended training requirements for becoming a specialist geriatrician within the EU [105]. Falls prevention was identified as a key area of knowledge in patient care [105]. We could not identify European undergraduate curriculums for physiotherapists, nurses, and occupational therapists, and it is likely that there is significant variation in the curriculum and the role of geriatrics and falls prevention across different European countries. It is essential that falls prevention is included in the undergraduate education of these healthcare professionals to ensure they are equipped with the necessary knowledge and skills.

In addition to the undergraduate education, the postgraduate dimension of geriatric medicine education is highly relevant, as most healthcare professionals, regardless of their speciality, will inevitably care for older patients and thus encounter issues related to falls [106]. However, geriatric syndromes are often not included in their generalist or speciality training [106]. This gap underscores the need to provide geriatric medicine competencies, including falls prevention, across all specialities, to ensure that healthcare professionals can effectively address the unique needs of older adults [106]. PROGRAMMING (PROmoting GeRiAtric Medicine in countries where it is still eMergING) COST Action focuses on advancing geriatric medicine (GM) education by defining and tailoring training for healthcare professionals, particularly in countries where GM is emerging, through identifying needs such as education on fall prevention, aligning global standards to local contexts, and leveraging expertise from established GM systems to enhance older adult care across diverse healthcare settings [107]. Since no Europe-wide programmes focused on fall prevention currently exist, there is a pressing need for multidisciplinary educational initiatives specifically based on the WFG to address this gap in training. Developing such initiatives would ensure a more consistent and comprehensive approach across the continent. Current European national educational programmes that are in place are listed in the Appendix for reference (Supplementary Table 3). Also, the American CDC has developed a free online course, STEADI: Empowering Healthcare Providers to Reduce Fall Risk [108].

A recent scoping review reported on education interventions for health professionals on falls prevention in healthcare settings [109]. They found that a variety of methods for education of health professionals in falls prevention has been investigated [109]. However, the authors underlined gaps in the planning, reporting, and evaluation processes for the healthcare professional education in fall prevention and recommend standardised reporting of education programmes [109].

The WFG also emphasises the importance of education for older adults, stressing that all older adults should be offered education on fall prevention regardless whether they are low-, intermediate-, or high-risk individuals. Resources for education are listed in Table 2. In the context of education, health literacy (HL), defined as an individual's capacity to obtain, comprehend, and use health-related information appropriately to maintain and enhance health, is particularly important [110]. The use and effectiveness of educational materials can vary depending on the participants' HL, making it a critical factor in the success of fall prevention efforts [110]. Furthermore, low HL impedes engaging interventions and using technological devices in implementing fall prevention [110]. However, it can be increased through tailored information, verbal debriefing, interactive communication, and culturally adapted interventions [110].

## Research

In the past, two European thematic networks, ProFaNE (Prevention of Falls Network Europe) and ProFouND (Prevention of Falls Network for Dissemination), played a pivotal role in bringing together key players across Europe [111]. These networks provided valuable resources and fostered collaboration among various stakeholders, many of whom were involved in the development of the WFG. In recent years, numerous attempts have been made through various European calls to secure funding for falls prevention research, but unfortunately, these efforts have not been successful. This underlines the need to connect with policymakers to re-prioritise the topic of fall prevention on the European political and research agenda. Currently, there is no established European-wide research agenda specifically addressing falls prevention and management among older adults. The absence of funding and a coordinated approach hinders the ability to effectively tackle this critical issue. Therefore, it is essential to create a comprehensive research agenda that outlines key priorities for investigation. In the following, we will discuss some of the items that we believe should be included in this research agenda.

The WFG itself reported several key areas for future research, e.g. frailty, sarcopaenia, technology, and implementation. Further research and consensus are necessary to refine the diagnostic and therapeutic approaches to sarcopaenia in individuals at risk of falls [7]. It remains unclear whether targeted interventions, such as exercise and protein supplementation, can effectively reduce fall risk in older adults with sarcopaenia [7]. Additionally, a deeper understanding is required regarding the integration of frailty into fall management strategies and its potential impact on fall risk reduction [7]. Frailty management shows promising potential, as in a recent longitudinal study, there was a lower risk of future falls in those who sustained frailty remission compared with those who remained frail and there is also emerging evidence on the effectiveness of physical activity interventions on the reduction of frailty, and the increase in muscle strength and physical performance [112], [113]. The working group on polypharmacy and fall-risk-increasing drugs of WFG outlined key items for a future research agenda in their position paper [114]. Furthermore, to facilitate the implementation of the WFG, it would be beneficial to conduct a health technology assessment of the WFG to provide valuable insights into cost-effectiveness, feasibility, and the potential impact of these interventions on the healthcare system [115].

A few retrospective evaluations using existing cohort data have been conducted regarding the new WFG risk stratification algorithm [116–119]. The algorithm successfully identifies those at greater risk of falling when using the opportunistic case-finding method [18–21]. When the Timed Up and Go test was utilised to differentiate between low and intermediate risk, the true value of the intermediate-risk group remained unclear due to the small number of individuals within this category [116, 118, 119]. Hicks et al. suggested two simple modifications to this algorithm, as they were able to identify a sizable intermediate-risk group that presented with physical and neuropsychological characteristics similar to the high-risk group, possibly indicating a medium- to long-term increase in their rate of falls with the modified algorithm [120]. These findings should be considered when the algorithm is updated to enhance clinical applicability. Also, a prospective study/simulation study is warranted to understand better the effects of risk stratification and the accompanying interventions [121]. Multifactorial prediction models are anticipated to deliver improved performance in fall prediction, albeit at the expense of usability, which warrants further research to enhance their practical application [121].

The 2005 Prevention of Falls Network Europe (ProFaNE) core outcome set (COS) identified key outcomes for falls prevention, including falls, injuries, psychological consequences of falling, generic health-related quality of life (HRQoL), and physical activity [122]. However, since the original publication, minimum standards for developing COS have been set [123]. Notably, people who had experienced falls did not contribute to developing the ProFaNE COS [122, 124]. Furthermore, two of the five ProFaNE COS domains (physical activity and HRQoL) were undefined, and one (physical activity) was not accompanied by a recommended measurement instrument set [122, 124]. Given these limitations, updating the ProFaNE COS is essential to align with contemporary standards and ensure that it encompasses a comprehensive and relevant set of outcomes. COS standardise the measurement and reporting of outcomes in research, ensuring consistency and enhancing the relevance of findings by incorporating stakeholder input [125]. This leads to improved quality of evidence, informed decision-making, and more efficient use of resources in evaluating interventions [125].

A recent scoping review on factors influencing the implementation of an exercise-based intervention to prevent falls in older community-dwelling individuals identified a relative lack of implementation research theory, evidence, and guidance informing community fall prevention exercise implementation [95]. Less than half of the articles reported an implementation strategy, and none reported using any implementation theory, model or framework to plan and design implementation [95]. In addition, there was substantial variation in reporting implementation outcomes and no outcome was consistently reported in all papers [95]. To address these gaps, it is essential to leverage and integrate implementation science more effectively in falls prevention research. The following steps are critical [126]:

i. *Clarify concepts*: Clearly define and differentiate between “evidence-based interventions” and “implementation strategies” in the context of fall prevention.
ii. *Contextual considerations*: Implementation success is highly dependent on the specific setting and circumstances in which it occurs. It is therefore crucial to identify, understand, and describe the contextual factors that influence the implementation process. Applying an implementation science framework can help systematically analyse these factors.
iii. *Standardise and include implementation outcomes*: Specify and evaluate key implementation outcomes for fall prevention. These outcomes should be consistently agreed upon across studies to allow for comparison and data synthesis, and to facilitate the translation of research findings into effective real-world practice.

Involving older adults and other stakeholders such as healthcare providers in tailoring the implementation strategy could increase the possibility of succeeding with implementation of falls prevention. Co-creation, where interventions are developed collaboratively with input from end users and stakeholders, is crucial in ensuring that falls prevention strategies are both relevant and effective in real-world settings. By integrating the perspectives of older adults, healthcare professionals, caregivers, and policymakers, interventions can be better tailored to meet the specific needs, preferences, and challenges faced by those at risk of falls. This participatory approach fosters greater ownership and acceptance of interventions, increasing the potential for adherence and long-term sustainability. This approach is also crucial for future research to ensure that interventions are effective, feasible, and responsive to the diverse needs of different healthcare professionals and systems and older adults.

Fall prevention strategies targeting disadvantaged groups of older adults are essential. Underserved groups, such as those living in deprived areas or with different ethnic backgrounds, or other protected characteristics such as individuals from lower socioeconomic backgrounds or older adults with limited social networks or community support, have often been overlooked in fall prevention research. These populations experience a higher prevalence of health problems, yet they probably remain underrepresented in studies and interventions aimed at reducing fall risks [127]. To address this gap, it is crucial to design tailored interventions that consider the specific needs, preferences, and barriers confronted by these underserved groups. By prioritising their inclusion in fall prevention efforts, we can enhance the effectiveness of interventions, promote equitable health outcomes, and ensure that all older adults, regardless of their background, have access to essential fall prevention resources and support. Furthermore, it is crucial to consider gender-specific risks, needs, and preferences when developing fall prevention strategies. Acknowledging the gender dimension will help ensure that interventions are more effective, inclusive, and responsive to the unique needs of both men and women. This consideration should be integrated into the design and implementation of fall prevention programmes to promote equitable outcomes for all.

## Conclusion

To implement falls prevention strategies successfully across Europe, the following collaborative actions are essential, ensuring a more unified and effective approach to falls prevention and management:

- *Align national and regional health policies with implementation goals*: Advocate for national and regional health policies that specifically support the implementation of fall prevention programmes.
- *Facilitate cross-sector partnerships for implementation*: Work across sectors, including healthcare, social services, and community organisations, to foster collaboration in implementing fall prevention strategies at the individual and population health levels.
- *Integrate fall prevention into broader healthcare policies*: Advocate for the inclusion of falls prevention in related policies such as healthy ageing, fracture prevention, frailty, public health, and emergency care to ensure a comprehensive approach to the care of older adults.
- *Engage policymakers*: Policymakers must be strongly encouraged to recognise the significant societal and economic impact of falls, so that they can prioritise fall prevention in public health agendas and allocate the necessary resources.
- *Create a European blueprint for fall prevention pathways*: A standardised but adaptable falls prevention care pathway is needed across Europe.
- *Develop tailored, evidence-based implementation plans*: Encourage the creation of local, evidence-based implementation plans that are customised to the specific healthcare system of each region.
- *Explore technology*: Understand the various technologies that can support and automate tasks within fall prevention.
- *Promote the use of implementation science*: Encourage the application of implementation science to identify effective strategies for integrating fall prevention into everyday clinical practice.
- *Incorporate older adult and community involvement in implementation efforts*: Ensure that the needs, priorities, and preferences of older adults are central to the implementation process.
- *Raise awareness among older adults and caregivers*: Public health campaigns and community programmes should actively inform older adults and caregivers about the risks of falls, prevention strategies, and available resources to empower them in taking proactive steps.
- *Educate healthcare professionals*: It is essential to ensure healthcare professionals are adequately trained in fall prevention. There is a need for a standardised under- and postgraduate curriculum dedicated to fall prevention across different healthcare professional groups.
- *Develop a European-wide research agenda*: There is a need for a coordinated research agenda addressing fall prevention and management, which should be supported by adequate funding.
- *Strengthen collaboration within the EuGMS SIG on Falls and Fractures*: Strengthening collaboration within the Special Interest Group (SIG) on Falls and Fractures will enhance knowledge dissemination, research, and the exchange of best practices across countries.

A commitment to these collaborative initiatives is essential for advancing fall prevention efforts across Europe. The Special Interest Group (SIG) on Falls and Fracture Prevention can play a key role in driving these initiatives. By prioritising these strategic actions, we can achieve substantial progress in mitigating the impact of falls and enhancing outcomes for older adults at risk of experiencing falls.

## Supplementary Information

Below is the link to the electronic supplementary material.

Supplementary file1 (DOCX 45 KB)
