# Supplementary material for: Falls prevention in community-dwelling older adults and implementation of world falls guidelines: a call for action across Europe by the European Geriatric Medicine Society Special Interest Group on Falls and Fractures
Source: Eur Geriatr Med. 2025 Jun 12;16(4):1249–68. doi: 10.1007/s41999-025-01206-y (PMC12378773; doi:10.1007/s41999-025-01206-y)
Supplement: Supplementary file 1 — Supplementary file1 (DOCX 45 KB) [file 41999_2025_1206_MOESM1_ESM.docx]

Supplementary Material

**Supplementary Table 1. Resources to Support Clinical Practice of Falls Prevention in English**

| Austria | "Trittsicher & aktiv" Program: (transl. "Sure-footed & Active") Offered by the Österreichische Gesundheitskasse (ÖGK), this program provides workshops and video series designed to improve balance, strength, and reaction time among older adults, thereby reducing the risk of falls. <https://www.gesundheitskasse.at/cdscontent/?contentid=10007.869494&portal=oegkportal> |
| --- | --- |
| Belgium | All relevant information can be found at valpreventie.be, more specific at: [www.valpreventie.be/aanbod](http://www.valpreventie.be/aanbod) |
| Czech Republic | <https://mzd.gov.cz/wp-content/uploads/wepub/18576/40360/NOP%20Prevence%20p%C3%A1d%C5%AF%20a%20postup%20p%C5%99i%20zran%C4%9Bn%C3%ADch%20zp%C5%AFsoben%C3%BDch%20p%C3%A1dy.pdf> |
| Denmark | A small clinical guideline at the website of the Danish geriatric society (Faldudredning)”: <https://geriatri.dk/forside/klinisk.php>  A recommendation from National Health board to the municipalities (2015) to address falls as part general preventive strategies: <https://www.sst.dk/da/Fagperson/Aeldres-sundhed-pleje-og-omsorg/Forebyggelse-blandt-%C3%A6ldre/Fald>  Healthy Cities Network has an aim to raise awareness and inspire prevention of falls among older adults: <https://sund-by-net.dk/temagrupper/faldforebyggelse/> |
| Finland | [The UKK Institute material](https://ukkinstituutti.fi/liikkumisen-turvallisuus/kaatumisten-ehkaisy-ammattilaisille/maailmanlaajuiset-suositukset-kaatumisten-ehkaisyyn/): a short webinar about the subject, a detailed translation of all the major domains of the 2022 WFG. |
| France | How to improve balance in older persons. Practical guide. [manuel_maintien_de_l_equilibre_anti_chutes_-_2023.pdf](https://sante.gouv.fr/IMG/pdf/manuel_maintien_de_l_equilibre_anti_chutes_-_2023.pdf)  Documents useful to implement the French fall prevention plan Blain H, Annweiler C, Berrut G, Bernard PL, Bousquet J, Dargent-Molina P, Friocourt P, Puisieux F, Robiaud JB, Rolland Y. Plan antichute des personnes âgées France 2022-2024 : objectifs et méthodologie1 [Anti-fall plan for the elderly in France 2022-2024: objectives and methodology]. Geriatr Psychol Neuropsychiatr Vieil. 2023 Sep 1;21(3):286-294. French. doi: 10.1684/pnv.2023.1122.  Blain H, Annweiler C, Berrut G, Bernard PL, Bousquet J, Dargent-Molina P, Friocourt P, Puisieux F, Robiaud JB, Rolland Y. Letter to the Editor: Launch of a 2022-2024 National Plan Against Falls in Older Persons in France. J Nutr Health Aging. 2023;27(4):309-310. doi: 10.1007/s12603-023-1902-1. |
| Greece | <https://heliost.gr/images/ESWTERIKOTEXT/ESWTERIKO6.pdf>  <https://www.heliost.gr/images/ESWTERIKOTEXT/MONOGRAFIA_21_NEW.pdf>  Dionyssiotis Y, Skarantavos G, Papagelopoulos P. Modern rehabilitation in osteoporosis, falls, and fractures. Clin Med Insights Arthritis Musculoskelet Disord. 2014 Jun 12;7:33-40. doi: 10.4137/CMAMD.S14077. PMID: 24963273; PMCID: PMC4064946. |
| Iceland | No material national or regional but for Landspitali University Hospital (the largest hospital in Iceland) there is an extensive material in Icelandic for falls and falls preventions in hospital, published in the quality improvement manual of the hospital. These documents are open to every health care professional. |
| Ireland | There is a national guideline on the review of patients (service users) who have fallen that has been published by the HSE. It is part of a Patient Safety and incident management initiative. Falls are documented as one of the most commonly reported incidents within the HSE and this document focuses on patient safety improvement to reduce the risk and recurrence of falls.  It is named -  Service user Falls: A Practical Guide for Review, Version 2, 2022, Incident Management Team, Quality and Patient Safety Directorate.  <https://www.hse.ie/eng/about/who/nqpsd/qps-incident-management/incident-management/service-user-falls-a-practical-guide-for-review.pdf>  It includes information on falls prevention and falls identification and assessment.  Rather than being a purely clinical guideline, it looks at the management of falls through the lens of incident management.  Prior to this there was a national strategy published by the HSE in 2008 “Strategy to Prevent Falls and Fractures in Ireland’s Ageing Population”. This includes Best practice guidelines but however has not been updated since 2008. The document is available at this link.  https://www.hse.ie/eng/services/publications/olderpeople/strategy-to-prevent-falls-and-fractures-in-irelands-ageing-population---full-report.pdf |
| Israel | The Ministry of Health, in collaboration with other organizations, established the National Fall Prevention Program with the aim of raising awareness about fall risks and introducing preventive and treatment measures: <https://me.health.gov.il/en/older-adult/specialist-advice/fall-prevention/how-to-prevent-falls/fall-prevention-national-program/> |
| Italy | To date, there is no updated document at national level. Due to the regional health organization, the focus on falls prevention is very heterogeneous and different documents have been provided at the national, regional, or local level. Some examples are reported below:   - <https://www.salute.gov.it/portale/documentazione/p6_2_2_1.jsp?id=1639> - <https://www.salute.gov.it/imgs/C_17_pubblicazioni_975_allegato.pdf> - <https://salute.regione.emilia-romagna.it/assistenza-ospedaliera/sicurezza-cure/le-raccomandazioni-regionali-1/linee-di-indirizzo-regionali-sulle-cadute-in-ospedale-2016/view> - <https://www.regione.umbria.it/documents/18/1261595/Bollettino+linee+cadute+dall%27alto.pdf/c1726f87-3412-45c1-9809-2404ff52b479> - <https://www.regione.lombardia.it/wps/wcm/connect/f6af4f98-18f9-4441-8550-20483ba95211/PRP_2021_2025_Lombardia.pdf?MOD=AJPERES&CACHEID=ROOTWORKSPACE-f6af4f98-18f9-4441-8550-20483ba95211-p1944uK> - <https://www.regione.lazio.it/sites/default/files/2021-03/Piano-regionale-prev-cadute-2016.pdf> - <https://www.sanita.puglia.it/documents/36057/307555/PROCEDURA%2BPER%2BLA%2BPREVENZIONE%2BE%2BLA%2BGESTIONE%2BDELLE%2BCADUTE%2BDEI%2BPAZIENTI%2B%28Regione%2BPuglia%2BPreocedura%2Bper%2Bla%2BPrevenzione%2Be%2Bla%2BGestione%2Bdelle%2BCadute%2Bdei%2BPazienti..pdf%29/06bc7a18-87a8-4e07-a6ff-9461ad8cc17d> - https://www.aslal.it/allegati/CRITERI_GUIDA_AZIENDALI_PER_LA_PREVENZIONE_DELLE_CADUTE_DEI_PAZIENTI_AFFERENTI_ALL_ASL_AL.pdf |
| Netherlands | The page from VeiligheidNL offers resources and information on fall prevention, focusing on reducing fall-related injuries among older adults:  [Kennisaanbod Valpreventie \| VeiligheidNL](https://www.veiligheid.nl/themas/valpreventie/kennisaanbod) |
| Poland | Specific training materials have been developed to support healthcare professionals in the clinical practice of falls prevention.  For example, materials for physiotherapists enrolled in the physiotherapy specilisation programme are provided in a series of training courses aimed at disseminating knowledge and reference materials on fall prevention in older adults. Likewise, material for medical specialisation of geriatrics. |
| Portugal | <https://normas.dgs.min-saude.pt/2019/12/09/prevencao-e-intervencao-na-queda-do-adulto-em-cuidados-hospitalares/>  Clinical recommendations to prevent falls in hospitalized patients, not limited to older adults. The development process was lengthy (working meetings began in 2016, based on international literature). Implementation has not been widely disseminated or standardized. |
| Slovenia | National institute for public health prepared special materials for falls prevention:  <https://nijz.si/wp-content/uploads/2022/07/padci_pri_starejsih_2019_publikacija_oblikovano_2020_koncna_mrs.pdf> |
| Spain | Update of the consensus document on prevention of frailty in elderly people (2022) . Annex 3 <https://www.sanidad.gob.es/areas/promocionPrevencion/envejecimientoSaludable/fragilidadCaidas/estrategiaSNS/docs/Update_of_the_consensus_document_on_prevention_of_frailty_in_elderly_people_2022_ACCESIBLE.pdf>  Evidence-based recommendations for the prevention and treatment of falls in older adults. Departamento de salud. Gobierno Vasco (2015) <https://www.euskadi.eus/contenidos/informacion/osteba_publicaciones/es_osteba/adjuntos/4_recomendaciones_prevenci%C3%B3n_caidas.pdf>  Other regional resources, what we have collected is here  <https://www.sanidad.gob.es/areas/promocionPrevencion/envejecimientoSaludable/enlacesDeInteres/EnlacesInteres_CCAA.htm> but there are not specific for falls |
| Sweden | The National Board of Health and Welfare’s platform for healthcare providers: <https://www.socialstyrelsen.se/kunskapsstod-och-regler/omraden/aldre/stod-i-arbetet--vard-och-omsorg-for-aldre-personer--utifran-amnesomraden/fallolyckor/>  The Handbook for Healthcare’s fall prevention materials: https://www.vardhandboken.se/vard-och-behandling/basal-och-preventiv-omvardnad/fallprevention/  The Region of Sörmland's educational platform for fall prevention for citizens and healthcare providers, collection of material and instruments to support clinical decision making: <https://www.fou.sormland.se/kunskapsportal/kunskapsstod-for-fallprevention/material-instrument-och-lankar/> |
| Turkey | The Ministry of Health website includes some informative documents:  https://shgmkalitedb.saglik.gov.tr/TR-105666/hasta-dusmelerinin-onlenmesi.html  https://hsgm.saglik.gov.tr/tr/yasli-sagligi/guvenli-cevre.html  https://shgmkalitedb.saglik.gov.tr/TR-105668/ilgili-olcekler.html  https://shgmkalitedb.saglik.gov.tr/TR-105669/ilgili-kaynaklar.html |
| UK | 1. UK College of Optometrists have a falls checklist for optometrists: [focus-on-falls-checklist.pdf](https://www.college-optometrists.org/coo/media/media/documents/falls/focus-on-falls-checklist.pdf) and <https://www.college-optometrists.org/category-landing-pages/falls/focus-on-falls> 2. The Royal College of Occupational Therapists have practice guidelines: <https://www.rcot.co.uk/practice-resources/rcot-practice-guidelines/falls> 3. The Royal college of physicians have a national audit of inpatient falls (<https://www.rcp.ac.uk/improving-care/national-clinical-audits/falls-and-fragility-fracture-audit-programme-fffap/national-audit-of-inpatient-falls-naif/>), a national falls and fragility fracture audit (<https://www.rcp.ac.uk/improving-care/national-clinical-audits/falls-and-fragility-fracture-audit-programme-fffap/>), care bundles (<https://rcp.ac.uk/media/3xcfcp5p/fallsafe-care-bundles.pdf>) 4. Chartered Society of Physiotherapy community guideline: <https://www.csp.org.uk/publications/physiotherapy-works-falls-community-approach> 5. National falls prevention group & pharmaceutical society: <https://www.rpharms.com/Portals/0/RPS%20document%20library/Open%20access/Pharmacy%20guide%20docs/Medicines%20and%20falls%209%2023%20%28RPSendorsed%29.pdf> 6. Scotland Patient Safety Programme – Falls Programme - <https://ihub.scot/improvement-programmes/acute-adult/falls/> 7. Wales – Age Cymru (charity) have a National Falls Prevention Taskforce - <https://www.agecymru.wales/our-work/promoting-health-and-wellbeing/falls/national-falls-prevention-taskforce-wales/#:~:text=Chaired%20by%20Age%20Cymru%2C%20and,private%20and%20third%20sector%20organisations>. 8. Northern Ireland Public Health Agency – Falls Prevention leadership - <https://www.publichealth.hscni.net/directorates/nursing-and-allied-health-professions/safety-and-quality/falls-prevention> |

**Supplementary Table 2.** Educational initiatives on Falls Prevention for Healthcare Professionals

| Austria | In Austria, continuing education and professional development for physiotherapists are offered by private training institutions, including:  <https://www.physio-zentrum.at/kurs/sturzpraevention/> |
| --- | --- |
| Belgium | The Centre of Expertise for Fall and Fracture Prevention Flanders has different training opportunities:   - - <https://www.valpreventie.be/vormingen>   Partner initiatives:   - REVAL Academy: [https://www.uhasselt.be/nl/studeren/opleidingen-professionals/reval-academy](https://www.google.com/url?q=https://www.uhasselt.be/nl/studeren/opleidingen-professionals/reval-academy&source=gmail-imap&ust=1732704961000000&usg=AOvVaw1_b0nKnibNuCoIL4WODVX8) - REKI connects: [https://www.uhasselt.be/nl/faculteiten/faculteit-revalidatiewetenschappen/nieuws/reki-connects-een-geslaagde-eerste-editie](https://www.google.com/url?q=https://www.uhasselt.be/nl/faculteiten/faculteit-revalidatiewetenschappen/nieuws/reki-connects-een-geslaagde-eerste-editie&source=gmail-imap&ust=1732704961000000&usg=AOvVaw1yzhCZoA-7i0IEYqeC5xLF) |
| Czech Republic | Czech Association of Nurses:  [zaverecna_zprava_2011_2012-91b6f.pdf](https://www.cnna.cz/docs/tiskoviny/zaverecna_zprava_2011_2012-91b6f.pdf)  [kdp-pady-plna-verze.pdf](https://dokumenty.osu.cz/lf/uom/uom-publikace/kdp-pady-plna-verze.pdf) |
| Denmark | Aim to increase awareness about falls prevention among healthcare workers produced by Healthy Cities Network: <https://sund-by-net.dk/publikationer/8-anbefalinger-til-forebyggelse-af-aeldres-faldulykker-den-gode-kommunale-model-revideret-2016/> |
| France | One of the goals of the SFGG falls prevention interest group will be to organize falls prevention teaching symposia during the annual French SFGG congress, to write teaching documents for geriatricians, MDs of other specialties, and non-medical health professionals, in order to disseminate the WFG. |
| Iceland | Material available for everyone:  <https://landspitali.is/byltur> |
| Ireland | For clinicans within the HSE there is an online learning platform called HSELand.  <https://www.hseland.ie/dash/Account/PreLogin>  On this platform there are links to classroom courses as well as online classes. The courses are written by and targeted at clinician groups including both public programmes and hospitals throughout Ireland.  The topics include prevention, assessment and management.  There are private courses available for healthcare professionals also.  This includes a “Falls prevention Training course” run by Safe Aid which is a company associated with the Irish Heart Foundation.  https://safeaid.ie/product/falls-prevention-training-course/#:~:text=On%20our%20Fall%20Prevention%20training,of%20care%20and%20support%20given.  There is also “Falls prevention training” run by a private company Fit for Life.  https://www.fitforlife.ie/falls-prevention-training |
| Italy | The available initiatives are heterogeneous among regional healthcare services. Some of them are mainly focused on falls occurring in hospital or long-term healthcare settings. A national document was issued in 2011 and requires revision.  The Italian Society of Gerontology and Geriatrics (SIGG) and the Fragility Fracture Network (FFN) in Italy are the main scientific bodies focusing on falls and offering some tools, seminars or symposia about updates on falls duting national and regional conferences (https://www.sigg.it/eventi-sigg-societa-italiana-gerontologia-geriatria/). |
| Netherlands | The VeiligheidNL webpage on fall prevention training provides information about programs designed to reduce fall injuries in older adults: [Opleidingen valpreventie \| VeiligheidNL](https://www.veiligheid.nl/opleidingen-valpreventie) |
| Poland | <https://www.cmkp.edu.pl/wp-content/uploads/2021/11/PROGRAM-SPECJALIZACJI-W-DZIEDZINIE-FIZJOTERAPII-aktualizacja-10.11.2021-1.pdf>  COURSE MODULE VIII Physiotherapy in the domain of psychiatry and geriatrics, spanning pages 38 – 45.  https://www.cmkp.edu.pl/wp-content/uploads/2023/02/0744-program-1-1.pdf |
| Portugal | Scientific societies have been organizing educational activities on fall prevention.  For example, the Geriatrics Study Group of the Portuguese Society of Internal Medicine organized a Falls Webinar in 2022: <https://www.spmi.pt/webinar-quedas-nos-idosos/>. The Annual Scientific Meeting usually includes discussions on fall-related topics. Additionally, some fall prevention-related literacy materials have been published and distributed to older adults.  The National Society of Orthopedics and Traumatology has also addressed fall prevention, focusing on the general population, with the campaign “Não Caia Nisso”: <https://spot.pt/campanhas-sociais/spot-relanca-campanha-nao-caia-nisso-para-prevenir-quedas-em-idosos/> |
| Spain | Training course Detection and management of frailty and falls in the elderly. <https://www.sanidad.gob.es/areas/promocionPrevencion/estrategiaSNS/planDeCapacitacion/caidasPersonasMayores.htm>  It is developed at the national level but is offered to the regions.  Vivifrail: Materials for professionals responsible for the prescription of a program of physical exercise to prevent weakness and falls in older people.  <https://vivifrail.com/resources/> |
| Sweden | The National Board of Health and Welfare’s platform for health care providers regarding fall prevention, Educational material: <https://kunskapsguiden.se/omraden-och-teman/aldre/fallolyckor-och-fallprevention/utbildningar/>  The Handbook for Healthcare’s fall prevention materials: https://www.vardhandboken.se/vard-och-behandling/basal-och-preventiv-omvardnad/fallprevention/  The Region of Sörmland's educational platform for fall prevention for citizens and healthcare providers: <https://www.fou.sormland.se/kunskapsportal/kunskapsstod-for-fallprevention/> |
| UK | 1. Most UK Health Trusts have mandatory falls training for clinical staff (either face to face or e-learning) 2. Royal College of Physicians (London) falls training: <https://www.rcp.ac.uk/improving-care/resources/fallsafe-and-carefall-e-learning/> 3. NHS Education for Scotland Falls, Frailty and Bone Health: Prevention and Management training for health and social care staff – available on TURAS (NHS Scotland workers have access). <https://learn.nes.nhs.scot/23390> 4. REACT TO FALLS training for care home staff - <https://reactto.co.uk/react-to-falls>   5. Later Life Training provide training in evidence based exercise (FaME - <https://laterlifetraining.co.uk/courses/postural-stability-instructor/about-postural-stability-instructor/> and Otago - <https://laterlifetraining.co.uk/courses/otago-exercise-programme-leader/about-otago-exercise-programme-leader/> ) |

**Supplementary Table 3.** Implementation guidance on Falls Prevention

| Austria | Guidelines:  There is a guideline for 'Fall Prevention in Older Adults in Hospitals and Long-Term Care Facilities' by the Medical University of Graz (Daniela Schoberer et al.) including section about implementation:  <https://pflegewissenschaft.medunigraz.at/frontend/user_upload/OEs/institute/pflegewissenschaft/pdf/EBN_Sturzleitlinie.pdf> |
| --- | --- |
| Belgium | The Centre of Expertise for Fall and Fracture Prevention Flanders has different implementation guides:   - Implementation plan for nursing homes:   - <https://storage.googleapis.com/smooty-1220.appspot.com/uploads/3124/1639406657_20211212ImplementatieplanvalenfractuurpreventieVlaamseWZCversieWZCFINAAL.pdf> - The BE-EMPOWERed implementation plan for home care:   - <https://www.valpreventie.be/be-empowered-pilootproject> |
| Czech Republic | NÁRODNÍ OŠETŘOVATELSKÝ POSTUP PREVENCE PÁDŮ A POSTUP PŘI ZRANĚNÍ ZPŮSOBENÝCH PÁDY  National nursing guideline of falls prevention and procedure for injuries caused by falls, Ministry of Health of the Czech Republic, 2020.  https://mzd.gov.cz/wp-content/uploads/wepub/18576/40360/NOP%20Prevence%20p%C3%A1d%C5%AF%20a%20postup%20p%C5%99i%20zran%C4%9Bn%C3%ADch%20zp%C5%AFsoben%C3%BDch%20p%C3%A1dy.pdf |
| Iceland | No material national or regional but for Landspitali University Hospital (the largest hospital in Iceland) there are videos on implementation of fall prevention programs on clinical wards.  <https://vimeo.com/706491646> |
| Ireland | Within the “Strategy to Prevent Falls and Fractures in Ireland’s Ageing Population” there is information on implementation of fall prevention strategies.  https://www.hse.ie/eng/services/publications/olderpeople/strategy-to-prevent-falls-and-fractures-in-irelands-ageing-population---full-report.pdf |
| Italy | The following document issued in 2009 is available: <https://www.salute.gov.it/imgs/C_17_pubblicazioni_975_allegato.pdf> |
| Netherlands | The "Handbook Fall clinics" provides guidance for establishing and evaluating fall clinics. It includes best practices, assessment tools, and protocols to enhance care for patients at risk of falling: [Handboek Valklinieken \| VeiligheidNL](https://www.veiligheid.nl/kennisaanbod/handleiding/handboek-valklinieken)  The "Ketenaanpak Valpreventie" is a structured strategy aimed at reducing fall risks among community-dwelling adults aged 65 and older: [Ketenaanpak Valpreventie \| VeiligheidNL](https://www.veiligheid.nl/ketenaanpak-valpreventie) |
| Spain | For exercise prescription in older persons with frailty and risk of falls  Vivifrail: physical training program to prevent weakness and falls in people over 70 years  <https://vivifrail.com/resources/> |
| Sweden | The National Board of Health and Welfare’s support for implementing work practices to prevent falls: <https://kunskapsguiden.se/omraden-och-teman/aldre/fallolyckor-och-fallprevention/infora-arbetssatt-for-att-forebygga-fallolyckor/> |
| UK | FaME toolkit – [Falls Management Exercise (FaME) Implementation Toolkit \| ARC EM 2024](https://arc-em.nihr.ac.uk/arc-store-resources/falls-management-exercise-fame-implementation-toolkit)  Raising the bar – Centre for Ageing Better – advice on implementing exercise programmes – <https://ageing-better.org.uk/resources/raising-bar-strength-balance> |
